# Supplementary material for: Improving RT-LAMP detection of SARS-CoV-2 RNA through primer set selection and combination
Source: PLoS One. 2022 Apr 1;17(4):e0254324. doi: 10.1371/journal.pone.0254324 (PMC8974972; doi:10.1371/journal.pone.0254324)
Supplement: S1 Table — All primers used in this study are shown here, with names as we refer to them and their original sources where appropriate. Amplicon size covers the F3-B3 distance as maps to the SARS-CoV-2 (MN908947.3), with positions on the genome listed. (PDF) [file pone.0254324.s001.pdf]

	Name in this study	Dong et al	Janikova et al	Name in or based on original paper	Primer	Sequence (5'-3')	SARS—CoV-2 Gene Target	Amplicon size (bp)	Amplicon location on MN908947	Reference
1	S2	S2	-	Lamb-ORF1a	F3	TCCAGATGAGGATGAAGAAGA	ORF1a	289	3043-3331	Lamb et al
					B3	AGTCTGAACAACTGGTGTAAG				
					FIP	AGAGCAGCAGAAGTGGCACAGGTGATTGTGAAGAAGAAGAG				
					BIP	TCAACCTGAAGAAGAGCAAGAACTGATTGTCCTCACTGCC				
					LF	CTCATATTGAGTTGATGGCTCA				
					LB	ACAAACTGTTGGTCAACAAGAC				
2	S12	S12	-	Broughton-E	F3	CCGACGACGACTACTAGC	E	234	26191-26424	Broughton et al
					B3	AGAGTAAACGTAAAAAGAAGGTT				
					FIP	CTAGCCATCCTTACTGCGCTACTCACGTTAACAATATTGCA				
					BIP	ACCTGTCTCTTCCGAAACGAATTTGTAAGCACAAGCTGATG				
					LF	TCGATTGTGTGCGTACTGC				
					LB	TGAGTACATAAGTTCGTAC				
3	S13	S13	-	Yang-E	F3	AGCTGATGAGTACGAACTT	E	216	26226-26441	Yang et al
					B3	TTCAGATTTTTAACACGAGAGT				
					FIP	ACCACGAAAGCAAGAAAAAGAAGTATTCGTTTCGGAAGAGACAG				
					BIP	TTGCTAGTTACACTAGCCATCCTTAGGTTTTACAAGACTCACGT				
					LF	n/a				
					LB	CTGCGCTTCGATTGTGTGCGT				
4	S14	S14		Jiang-N	F3	CCAGAATGGAGAACGCAGTG	N	216	28354-28569	Jiang et al
					B3	CCGTCACCACCACGAATT				
					FIP	AGCGGTGAACCAAGACGCAGGGCGCGATCAAAACAACG				
					BIP	AATTCCCTCGAGGACAAGGCGAGCTCTTCGGTAGTAGCCAA				
					LF	TTATTGGGTAAACCTTGGGGC				
					LB	TTCCAATTAACACCAATAGCAGTCC				
5	S17	S17		Park-N_21	F3	GCCAAAAGGCTTCTACGCA	N	198	28774-28971	Park et al (N_21)
					B3	TTGCTCTCAAGCTGGTTCAA				
					FIP	TCCCCTACTGCTGCCTGGAGGCAGTCAAGCCTCTTCTCG				
					BIP	TCTCCTGCTAGAATGGCTGGCATCTGTCAAGCAGCAGCAAAG				
					LF	TGTTGCGACTACGTGATGAGGA				
					LB	ATGGCGGTGATGCTGCTCT				
6	Joung			Joung-N	F3	GCTGCTGAGGCTTCTAAG	N	326	29024-29349	Joung et al
					B3	GCGTCAATATGCTTATTCAGC				
					FIP	GCGGCCAATGTTTGTAATCAGTAGACGTGGTCCAGAACAA				

					BIP	TCAGCGTTCTTCGGAATGTCGCTGTGTAGGTCAACCACG				
					LF	CCTTGTCTGATTAGTTCCTGGT				
					LB	TGGCATGGAAGTCACACC				
7	S18	S18	N-Lu	Lu-N	F3	GCCAAAAGGCTTCTACGCA	N	198	28774-28971	Lu et al (added LF in this work)
					B3	TTGCTCTCAAGCTGGTTCAA				
					FIP	TCCCCTACTGCTGCCTGGAGCAGTCAAGCCTCTTCTCGTT				
					BIP	TCTCCTGCTAGAATGGCTGGCATCTGTCAAGCAGCAGCAAAG				
					LF	TCTTGAACTGTTGCGACTAC				
					LB	TGGCGGTGATGCTGCTCTT				
8	N2			Zhang-N2	F3	ACCAGGAACTAATCAGACAAG	N	188	29136-29323	Zhang et al
					B3	GACTTGATCTTTGAAATTTGGATCT				
					FIP	TTCCGAAGAACGCTGAAGCGGAACTGATTACAAACATTGGCC				
					BIP	CGCATTGGCATGGAAGTCACAATTTGATGGCACCTGTGTA				
					LF	GGGGGCAAATTGTGCAATTTG				
					LB	CTTCGGGAACGTGGTTGACC				
9	S4	S4		Park-Nsp3_2-24	F3	TGCAACTAATAAAGCCACG	ORF1a	194	6253-6446	Park et al (Nsp3_2-24)
					B3	CGTCTTTCTGTATGGTAGGATT				
					FIP	TCTGACTTCAGTACATCAAACGAATAAATACCTGGTGTATACGTTGTC				
					BIP	GACGCGCAGGGAATGGATAATTCCACTACTTCTTCAGAGACT				
					LF	TGTTTCAACTGGTTTTGTGCTCCA				
					LB	TCTTGCCTGCGAAGATCTAAAAC				
10	S10	S10		Park-S_1-2-2	F3	CTGACAAAGTTTTCAGATCCTCAG	S	209	21678-21886	Park et al (S_1-2-2)
					B3	AGTACCAAAAATCCAGCCTCTT				
					FIP	TCCCAGAGACATGTATAGCATGGAATCAACTCAGGACTTGTTCTTACC				
					BIP	TGGTACTAAGAGGTTTGATAACCCTGTTAGACTTCTCAGTGGAAGCA				
					LF	CCAAGTAACATTGGAAAAGAAA				
					LB	GTCCTACCATTTAATGATGGTGTTT				
11	E1			Zhang-E1	F3	TGAGTACGAACTTATGTACTCAT	E	210	26232-26441	Zhang et al
					B3	TTCAGATTTTTAACACGAGAGT				
					FIP	ACCACGAAAGCAAGAAAAAGAAGTTCGTTTCGGAAGAGACAG				
					BIP	TTGCTAGTTACACTAGCCATCCTTAGGTTTTACAAGACTCACGT				
					LF	GCGCTTCGATTGTGTGCGT				
					LB	CGCTATTAACTATTAACG				
12	As1e		As1e	Rabe-Asle	F3	CGGTGGACAAATTGTCAC	ORF1a	197	2245-2441	Rabe et al
					B3	CTTCTCTGGATTTAACACACTT				
					FIP	TCAGCACACAAAGCCAAAAATTTATTTTTCTGTGCAAAGGAAATTAAGGAG				

					BIP	TATTGGTGGAGCTAAACTTAAAGCCTTTTCTGTACAATCCCTTTGAGTG				
					LF	TTACAAGCTTAAAGAATGTCTGAACACT				
					LB	TTGAATTTAGGTGAAACATTTGTCACG				
13	N-Baek		Baek et al (N gene)	Baek-N	F3	TGGACCCCAAAATCAGCG	N	245	28285-28529	Baek et al
					B3	AGCCAATTTGGTCATCTGGA				
					FIP	CGTTGTTTTGATCGCGCCCCATTACGTTTGGTGGACCCTC				
					BIP	ATACTGCGTCTTGGTTCACCGCATTGGAACGCCTTGTCCTC				
					LF	TGCGTTCTCCATTCTGGTTACT				
					LB	TCTCACTCAACATGGCAAGGAA				
14	SGF-wt			SGF-wt	F3	TTCTCTTGCCACTGTAGC	ORF1a	207	11191-11397	This work
					B3	AGTGTCCACACTCTCCTAG				
					FIP	CCAGACAAACTAGTATCAACCATTCTATATGCCTGCTAGTTGG				
					BIP	GTTTTAAGCTAAAAGACTGTGTTATGGTTCTTGCTGTCATAAGGATT				
					LF	CCAACCATGTCATAATACGCATC				
					LB	ATGCATCAGCTGTAGTGTTACT				
15	S11	S11	Yan et al (S gene S-123)	Yan-S-123	F3	TCTATTGCCATACCCACAA	S	245	23693-23937	Yan et al
					B3	GGTGTTTTGTAAATTTGTTTGAC				
					FIP	CATTCAGTTGAATCACCACAAATGTGTGTTACCACAGAAATTCTACC				
					BIP	GTTGCAATATGGCAGTTTTTGTACATTGGGTGTTTTTGTCTTGTT				
					LF	ACTGATGTCTTGGTCATAGACACT				
					LB	TAAACCGTGCTTTAACTGGAATAGC				
16	S-Huang		Huang etal (S gene S17)	Huang-S17	F3	TCTTTCACACGTGGTGTT	S	233	21653-21885	Huang et al
					B3	GTACCAAAAATCCAGCCTC				
					FIP	CATGGAACCAAGTAACATTGGAAAACCTGACAAAGTTTTCAGATCC				
					BIP	CTCTGGGACCAATGGTACTAAGAGGACTTCTCAGTGGAAGCA				
					LF	GAAAGGTAAGAACAAGTCCTGAGT				
					LB	CTGTCCTACCATTTAATGATGGTGT				
17	Mam-N	S19		Broughton-N	F3	AACACAAGCTTTCGGCAG	N	229	29083-29311	Broughton et al
					B3	GAAATTTGGATCTTTGTCATCC				
					FIP	TGCGGCCAATGTTTGTAATCAGCCAAGGAAATTTTGGGGAC				
					BIP	CGCATTGGCATGGAAGTCACTTTGATGGCACCTGTGTAG				
					LF	TTCCTTGTCTGATTAGTTC				
					LB	ACCTTCGGGAACGTGGTT				
18	Lau			N-Lau	F3	GTTGTTCGTTCTATGAAGACT	N	200	28195-28394	Lau et al
					B3	GACGTTGTTTTGATCGCG				
					FIP	TGGGGTCCATTATCAGACATTTTAG TTTTAGAGTATCATGACGTTCG				

					BIP	CGAAATGCACCCCGCATTACCCAC TGCGTTCTCCATTC				
					LF	TGTTCGTTTAGATGAAATC				
					LB	TGGTGGACCCTCAGATTCAA				
